# Predicting epidermal growth factor receptor gene amplification status in glioblastoma multiforme by quantitative enhancement and necrosis features deriving from conventional magnetic resonance imaging

**DOI:** 10.1097/MD.0000000000010833

**Published:** 2018-05-25

**Authors:** Fei Dong, Qiang Zeng, Biao Jiang, Xinfeng Yu, Weiwei Wang, Jingjing Xu, Jinna Yu, Qian Li, Minming Zhang

**Affiliations:** a Department of Radiology; b Department of Neurosurgery; c Department of Pathology, The Second Affiliated Hospital, Zhejiang University School of Medicine, Hangzhou; d Department of Radiology, Shaoxing Second Hospital, Shaoxing, China.

**Keywords:** epidermal growth factor receptor, glioblastoma multiforme, magnetic resonance imaging

## Abstract

To study whether some of the quantitative enhancement and necrosis features in preoperative conventional MRI (cMRI) had a predictive value for epidermal growth factor receptor (EGFR) gene amplification status in glioblastoma multiforme (GBM).

Fifty-five patients with pathologically determined GBMs who underwent cMRI were retrospectively reviewed. The following cMRI features were quantitatively measured and recorded: long and short diameters of the enhanced portion (LDE and SDE), maximum and minimum thickness of the enhanced portion (MaxTE and MinTE), and long and short diameters of the necrotic portion (LDN and SDN). Univariate analysis of each feature and a decision tree model fed with all the features were performed. Area under the receiver operating characteristic (ROC) curve (AUC) was used to assess the performance of features, and predictive accuracy was used to assess the performance of the model.

For single feature, MinTE showed the best performance in differentiating EGFR gene amplification negative (wild-type) (nEGFR) GBM from EGFR gene amplification positive (pEGFR) GBM, and it got an AUC of 0.68 with a cut-off value of 2.6 mm. The decision tree model included 2 features MinTE and SDN, and got an accuracy of 0.83 in validation dataset.

Our results suggest that quantitative measurement of the features MinTE and SDN in preoperative cMRI had a high accuracy for predicting EGFR gene amplification status in GBM.

## 1 Introduction

Glioblastoma multiforme (GBM) is a World Health Organization grade IV glioma and represents the most common and aggressive type of primary brain tumor.^[1]^ GBM accounts for 45.2% of all malignant central nervous system (CNS) tumors, 80% of all primary malignant CNS tumors, and approximately 54.4% of all malignant gliomas.^[2]^ Even with efforts of various combined treatments, including surgical resection, radiotherapy and chemotherapy, the median survival period of GBM patients remains less than 15 months.^[3–5]^ Recent advances in genomic technologies have led to a deeper understanding of the key genetic alterations that underlie GBM, and these advances have enabled the development of more effective patient stratification, targeted therapeutics, and predictions of patient outcomes.^[6]^

The most frequent type of genetic alteration involves the epidermal growth factor receptor (EGFR), which is altered in approximately 50% of GBM patients. EGFR is a type of receptor tyrosine kinase (RTK), the activation of which results in the activation of multiple downstream signal transduction pathways such as the PI3K/Akt/mTOR pathway.^[7]^ EGFR plays a central role in cell division, migration, adhesion, differentiation and apoptosis,^[8,9]^ which together influence survival, motility, invasiveness, and resistance to treatment.^[10–12]^ Detecting the status of EGFR aberrations is helpful for classifying the molecular subtypes, evaluating the treatment effects and predicting the prognoses of GBM cases.^[2]^ In addition, the evaluation of EGFR amplification status may be of particular value for selecting patients for targeted therapy.^[13]^

Based on the role of EGFR in GBM, we hypothesized that EGFR gene amplification status is closely related to the size of the enhancement and necrosis portions of tumors observed in conventional MR images and that EGFR gene amplification status may be directly differentiated by using conventional MRI features. The goal of this study was to explore whether some enhancement and necrosis features by quantitatively measuring in preoperative conventional MR images had a predictive value for EGFR gene amplification status in GBM.

## 2 Materials and methods

This retrospective study was approved by the Local Ethics Committee of the Second Affiliated Hospital of the Zhejiang University School of Medicine; patient approval or informed consent for the review of patient images was not required. Informed consent for EGFR gene evaluation was obtained at the time of surgery.

### 2.1 Patients

This study included 55 patients with GBM who underwent surgical treatment at our institution between January 2015 and February 2016. The inclusion criteria were as follows: availability of presurgical MRI scans, including T1-weighted, T2-weighted, and postcontrast T1-weighted images; surgery performed within 1 week following MR scanning; pathologically confirmed GBM; and known EGFR amplification status that was determined using fluorescence in situ hybridization (FISH). Patients with secondary GBM were not included. Patient records/information was anonymized and de-identified prior to analysis.

### 2.2 Conventional MRI parameters

MR imaging was performed with 1.5 T (Signa Excite, GE Healthcare, Milwaukee, Wisconsin) and 3 T magnets (HDxt; Discovery 750; GE Healthcare, Milwaukee, Wisconsin). We acquired all images using a 6-mm section thickness. The preoperative imaging protocol consisted of axial T1-weighted and T2-weighted images, as well as contrast coronal, sagittal, and axial T1-weighted images. Gadodiamide (Omniscan, GE Healthcare, Co. Cork, Ireland) was injected though a peripheral venous catheter at a dose that was standardized based on patient body weight (0.2 mL/kg body weight, up to a maximum of 20 mL). The same dose of contrast was administered for both the 1.5 and 3 T scans.

### 2.3 Imaging analysis

**Figure 1:**
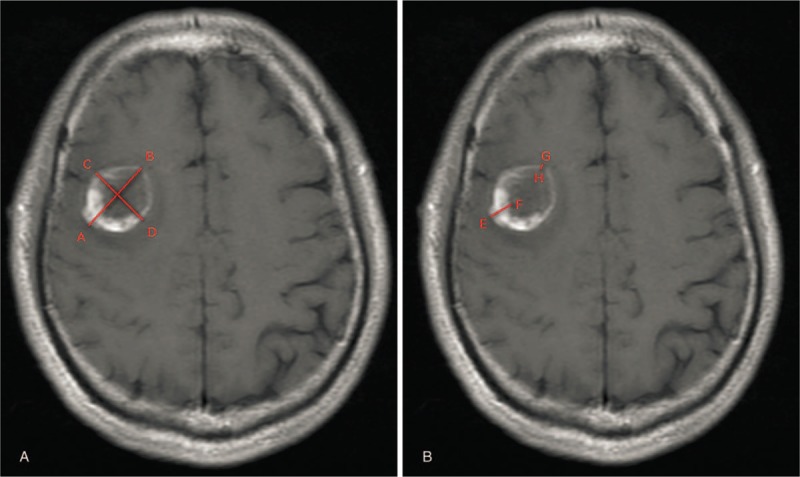
Example of an image for diameter and thickness measurement in cMRI (enhanced T1WI). (A) Line AB and line CD represent LDE and SDE, respectively. The LDN and SDN are measured in the same way as LDE and SDE but in the necrosis portion. (B) Line EF and Line GH represent the MaxiTE and MinTE, respectively. cMRI = conventional magnetic resonance imaging, LDE = long diameter of the enhanced portion, LDN = long diameter of the necrotic portion, MaxTE = maximum thickness of the enhanced portion, MinTE = minimum thickness of the enhanced portion, SDE = short diameter of the enhanced portion, SDN = short diameter of the necrotic portion.

Two radiologists (QL and FD), who were blinded to the patients’ EGFR amplification status, independently analyzed the MR images on a standard picture archiving and communication system. The following conventional MR imaging features were assessed or measured: LDE and SDE, MaxTE and MinTE, and LDN and SDN. The necrotic portion was defined as an irregular region that was surrounded by enhanced portion and that usually showed hyperintensity in the T2-weighted images. The maximum and minimum thicknesses were defined as the maximum and minimum thicknesses of the enhanced portion measured on the axial, sagittal, or coronal enhanced T1-weighted images. The long and short diameters were defined as the maximum perpendicular diameter measured on the axial, sagittal, or coronal-enhanced T1-weighted images (Fig. 1). The mean value of each feature got from the 2 measurers was recorded.

### 2.4 EGFR analysis

EGFR gene amplification status was determined using interphase/nuclear FISH on paraffin sections. For each tissue sample, a total of 200 cells were analyzed, and the results were reported as the highest level of EGFR amplification in chromosome copies per cell. EGFR amplification was defined as≥10 copies per cell, EGFR wild-type was defined as < 10 copies per cell.^[14]^

### 2.5 Data processing and analysis

For each of the feature, ROC curve was performed, and AUC value was computed.

Two datasets were set up using the primary data. Forty-five cases were used as training dataset to build a decision tree model with all of the features, and the rest 10 cases served as a validation dataset. Cases were randomly distributed to the 2 datasets.

To build a decision tree model with less complexity, pruning technique was used. And tenfold cross-validation method was also implemented in the process. The 10-fold cross-validation method involves partitioning the training dataset into 10 equal-sized subsamples randomly. Nine subsamples are then served as new training dataset, and the remaining subsample is used as the validation dataset for testing the model. The cross-validation process was repeated 10 times. The minimum “xerror” value based on 10-fold cross-validation were used to select the parameter “cp”(complexity parameter) for building the decision tree model. The model was at last validated with the validation dataset. Accuracy was used to assess the performances of the model.

All of the data processing, analysis and graphics were performed using R 3.3.1 (http://www.Rproject.org). The following R packages were used: “caret,” “rpart,” “Daim,” “DMwR.”

## 3 Results

### 3.1 Patients

This study included 55 patients (35 men and 20 women) with 55 GBMs. Patient ages ranged between 11 and 79 years, with a mean of 54 years. All tumors were treated with surgical resection. Using FISH technique, we detected pEGFR in 17 GBMs, while nEGFR in 38 GBMs.

### 3.2 Performance of features and models

**Figure 2:**
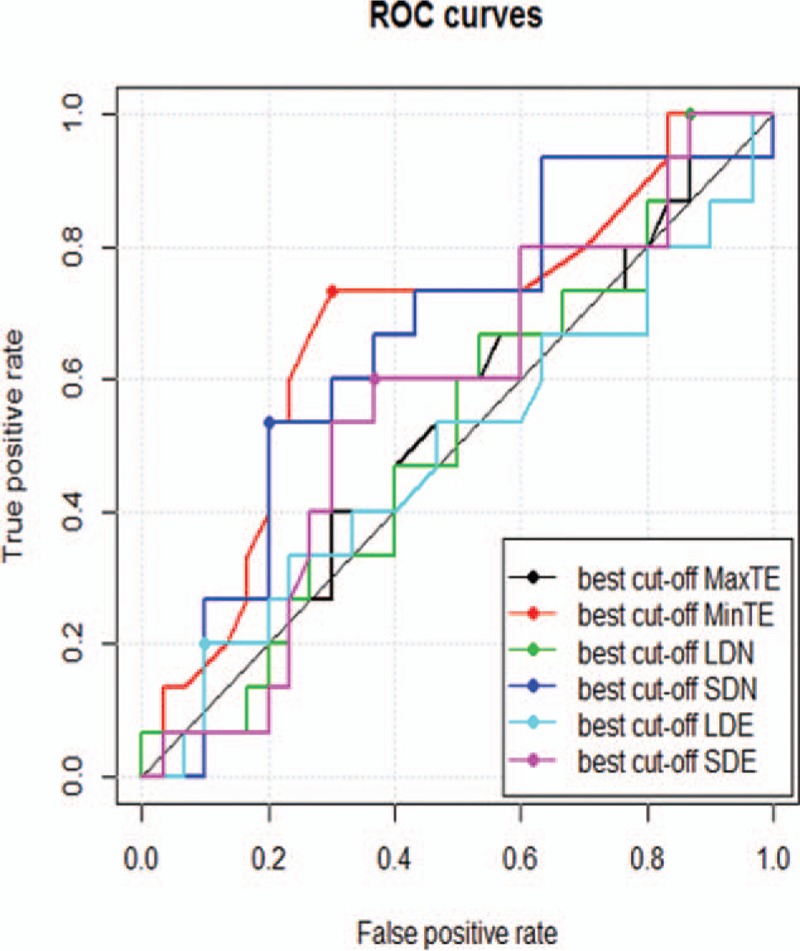
ROC curves for the differentiation of EGFR gene amplification status with single feature. The best feature for differentiating pEGFR GBM from nEGFR was MinTE, with a cut-off value of 2.6 mm and an AUC of 0.68. AUC = area under the curve, EGFR = epidermal growth factor receptor, GBM = glioblastoma multiforme, nEGFR = EGFR gene amplification negative, MinTE = minimum thickness of the enhanced portion, ROC = receiver operating characteristic.

The baseline MRI features are shown in Table 1. For each feature, the ROC curve is shown in Figure 2. The performance of each feature was listed in Table 2.

**Table 1:**
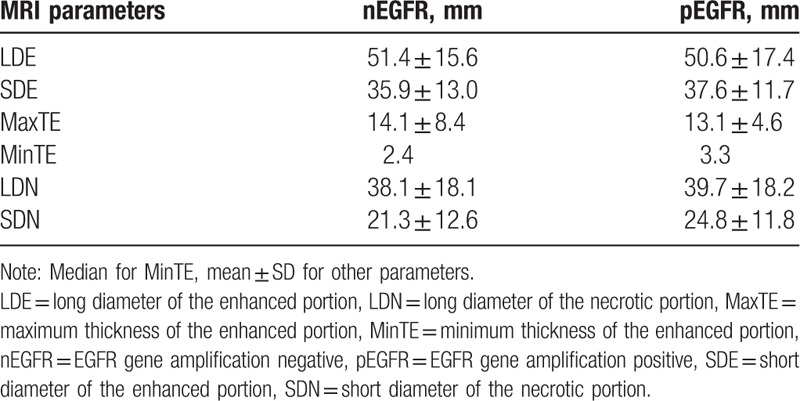
Baseline conventional MRI features and EGFR gene amplification status.

**Table 2:**
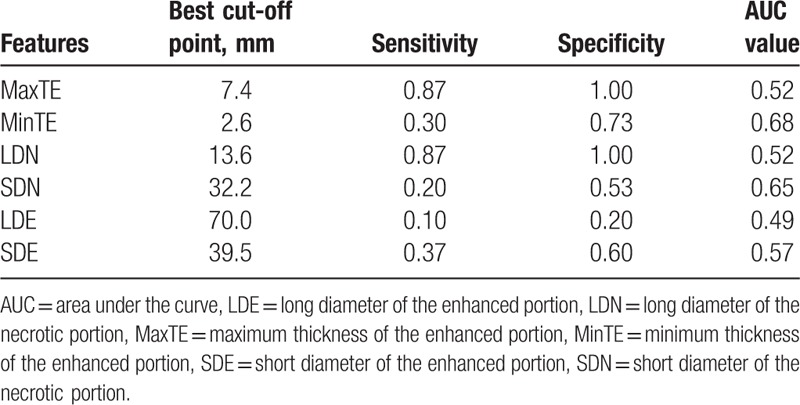
The performance of each MR feature.

**Figure 3:**
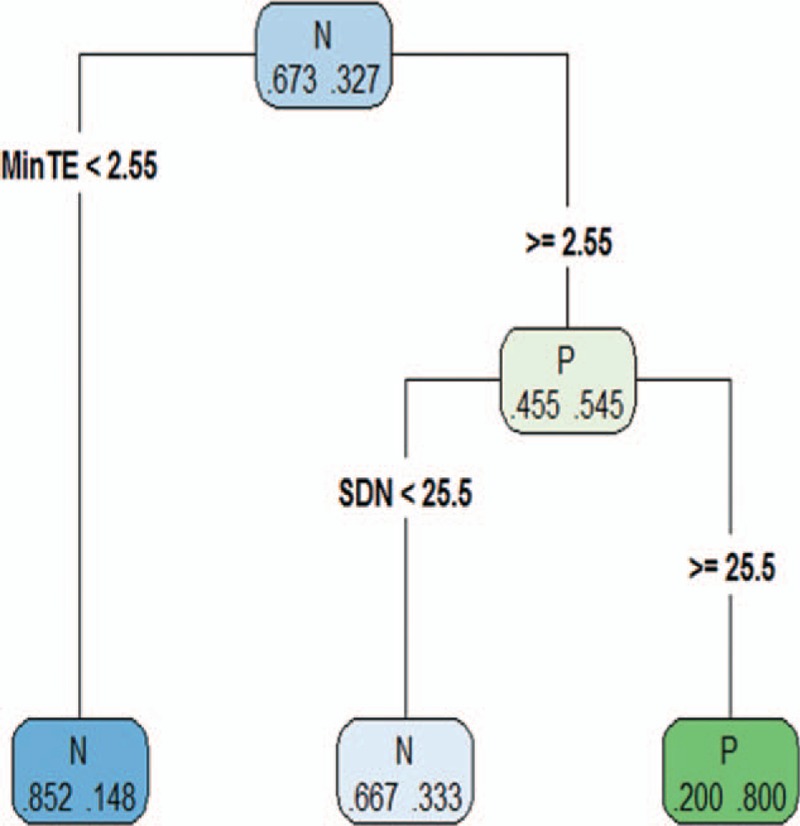
Decision tree model. For each node, the frame shows the larger proportion class (N or P) and the probability of per class in the node (left for N, right for P). Two branches, which derive from the nodes according to the value of features, stand for the classification. The predictive accuracy of the model was about 80% for training dataset, and 83% for validation dataset. GBM = glioblastoma multiforme, MinTE = minimum thickness of the enhanced portion, nEGFR = EGFR gene amplification negative, pEGFR = EGFR gene amplification positive.

Based on 10-fold cross-validation, the minimum “xerror” was 0.94, and a “cp” value of 0.01 with “nsplit” value of 2 were selected for building the model (Fig. 3). The decision tree model included 2 features MinTE and SDN. The predictive accuracy of the model was 0.80 for training dataset, and 0.83 for the validation dataset (Table 3)

**Table 3:**
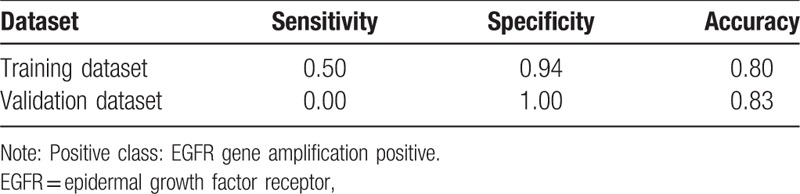
The performance of decision tree model.

## 4 Discussion

MRI has the potential to noninvasively provide important information for the diagnosis, treatment and evaluation of GBM. In this study, we evaluated some enhancement and necrosis features, as derived from cMRI, for GBM, and we investigated whether these features were predictive of EGFR gene amplification status in GBM.

We found that the single feature showed a weak value for differentiating EGFR gene amplification status in GBM. When combined with the features MinTE and SDN, it got a high accuracy for predicting EGFR gene amplification status in GBM.

A prior study has evaluated the ability of cMRI to predict EGFR gene amplification,^[14]^ with the results showing that none of the included cMRI features, such as border sharpness, cystic/necrotic change, hemorrhage, T2-isointense signal, nodular enhancement, subependymal enhancement, and multifocal discontinuous enhancement, demonstrated an ability to predict EGFR gene amplification status. The main difference between their features and ours was that all the cMRI features were nonquantitative in their study, whereas all of our imaging features were quantitative and easily accessible in work setting.

All of the single features in our study showed a limited value for differentiating EGFR gene amplification status in GBM, and the maximum AUC value of 0.68 came from feature MinTE. The prior study got a similar result that the most valuable single feature apparent diffusion coefficient showed an AUC value of 0.68.^[14]^ This might indicate that single feature had some difficulty in differentiating EGFR gene amplification status in GBM. The reason might be explained by imaging feature and EGFR gene expression was not one to one correspondence, as the signal signaling pathway was complicated in GBM.^[3,6,7]^

The models in our study showed a high predictive accuracy of EGFR gene amplification status in GBM by using only 2 features MinTE and SDN. MinTE is a feature of enhancement, and SDN represent a feature of necrosis. Though enhancement and necrosis each might be influenced by EGFR gene expression,^[3,15,16]^ it was hard to explore the exact relationship between EGFR gene amplification status and MinTE and SDN, as enhancement and necrosis were correlated with each other themselves and some of the mechanism of necrosis remains unclear.^[17]^

Some potential applications are suggested by our findings, as our model had a high predictive accuracy for EGFR gene amplification status. Being able to predict EGFR gene amplification status in patients with GBM may enable the prediction of the classical subtype of GBM, in which approximately 97% of cases show EGFR gene amplification. Furthermore, being able to predict EGFR gene amplification status is beneficial for making decisions about treatment and for the evaluation of disease prognosis.^[9,14]^ Predicting EGFR gene amplification status may be also useful for selecting candidates for EGFR-targeted drugs.

Several limitations should be considered for the current study. First, our study included a small number of subjects and the data were imbalanced, and prospective confirmation of these results with a larger sample size is needed. Second, though the EGFR analysis method is wildly used in standard clinical and research practices,^[14]^ heterogeneity of EGFR gene expression in the tumor may influence the results.

## 5 Conclusion

Our study found that by quantitative measuring the features MinTE and SDN in preoperative cMRI and using the decision tree model, EGFR gene amplification status in GBM could be noninvasively predicted with a high accuracy.

## Author contributions

**Data curation:** Fei Dong, Qian Li, Minming Zhang.

**Formal analysis:** Fei Dong, Qiang Zeng, Weiwei Wang, Jingjing Xu, Qian Li, Minming Zhang.

**Investigation:** Fei Dong, Qiang Zeng, Biao Jiang, Jingjing Xu, Qian Li.

**Methodology:** Fei Dong, Biao Jiang, Xinfeng Yu, Weiwei Wang, Jinna Yu, Qian Li.

**Project administration:** Fei Dong, Qian Li, Minming Zhang.

**Resources:** Fei Dong, Qiang Zeng, Weiwei Wang, Jinna Yu.

**Software:** Fei Dong, Xinfeng Yu.

**Validation:** Fei Dong, Jinna Yu, Qian Li.

**Visualization:** Fei Dong, Qian Li.

**Writing – original draft:** Fei Dong, Qian Li.

**Conceptualization:** Biao Jiang, Jingjing Xu, Qian Li, Minming Zhang.

**Writing – review & editing:** Jingjing Xu, Qian Li.

**Supervision:** Qian Li, Minming Zhang.
